# Temperament and Academic Achievement in Children: A Meta-Analysis

**DOI:** 10.3390/ejihpe11030053

**Published:** 2021-07-12

**Authors:** Dalia Nasvytienė, Tomas Lazdauskas

**Affiliations:** Institute of Psychology, Faculty of Philosophy, Vilnius University, 3 Universiteto Str., LT-01513 Vilnius, Lithuania; tomas.lazdauskas@fsf.vu.lt

**Keywords:** academic achievement, meta-analysis, temperament

## Abstract

This study aimed to systematize the diverse and rather controversial findings of empirical research on the relationship between the temperament and academic achievement of school children, as well as to determine the average effect size between these variables. We included 57 original studies of published and unpublished research conducted in 12 countries between 1985 and 2019, with cumulative sample size of 79,913 (varying from 6333 to 14,126 for links between particular temperament dimensions and specific domains of achievement). A random-effects and mixed-effects model was fitted to the data for the central tendency of the temperament–achievement relation and for analyzing moderators, respectively. The high heterogeneity of studies was tackled by selected specific moderators, namely, education level, transition status, family’s socio-economic level, and sources of report on achievement and temperament. The main findings of this meta-analysis affirmed the positive association of effortful control (EC) and inverse relationship of negative affectivity (NA) with a child’s academic performance, together with no apparent trend of surgency (SU) in this relationship; additionally, the sources of report significantly moderated the link between temperament and academic achievement.

## 1. Introduction

### 1.1. Interface between Temperament and Academic Achievement

A comprehensive explanation of variance in academic achievement encompasses cognitive and non-cognitive variables [1,2,3]. The latter refers to the wide realm of personality-related attributes, including temperament. Existing meta-analytic reviews have focused on only one temperamental category [4,5], while secondary analyses included only a few temperament-related characteristics as non-cognitive predictors of academic achievement [6,7]. Several studies have reported that many facets of temperament contribute jointly to school performance within an educational context [8,9,10,11]. To the best of our knowledge, the effect of various temperamental categories on children’s academic achievement has not yet been summarized.

Temperament is the earliest emerging characteristic within an individual and is relatively stable over the school years. It predisposes the child to interact with the environment in a particular way, and it is consistent across situations. Temperament was conceptualized in many ways such as: (1) a child’s behavioral style [12], (2) individual differences in reactivity and self-regulation [13], (3) inhibited or uninhibited response to novelty [14], and (4) patterns of emotionality, activity, and sociability [15]. Considering the codependency of children’s cognitions and emotions [16], it can be assumed that children’s temperaments affect their learning and its outcomes; moreover, as per Keogh [17], it accounts for a child’s ability to use what they know. Thus, it may suggest the important role of temperament—among all non-cognitive determinants—in academic achievement, which refers to “a level of proficiency in scholastic work in general or in a specific skill, such as arithmetic or reading” [18] (p. 5).

Temperamental attributes in the educational context have been explored for almost a century. There have been supportive opinions regarding its involvement in students’ academic success [19,20,21,22,23] as well as skeptical ones [24,25]. However, this particular research was conducted with college and university students. Systematic empirical studies on temperament in the school context started in the early 1980s. Keogh et al. [26] extensively supported the hypothesis that teachers’ academic decisions were influenced by their perceptions of pupils’ temperament, especially in cases of limited cognitive or physical development. They investigated how characteristics of children’s temperament (e.g., task orientation, flexibility, and reactivity) influenced teachers’ perception of students as “more teachable” or “less teachable”. Similarly, Lerner and Lerner [27] found that temperamental fit with the educational demands led to higher academic achievement. Furthermore, Martin and Holbrook [28] conducted a study that clearly showed how prediction of achievement by temperament (activity, distractibility, and persistence) exceeded that by intelligence. Meanwhile, Maziade et al. [29] found some evidence in support of temperament’s relationship with achievement, and Talwar et al. [30] reported indirect effects between temperament and academic skills. These studies were based on the interactional model of temperament, which was developed in the New York Longitudinal Study (NYLS) by Thomas and Chess with colleagues [12].

In recent times, most studies have been based on the developmental approach to temperament. It was introduced and elaborated by Rothbart and her colleagues [13,31,32]. According to it, temperament characteristics consistently fall into three dimensions. Effortful control (EC) is featured by regulatory skills in motor and cognitive domains, manifested mainly through inhibitory control, attention focus and shifting, and perceptual sensitivity. Negative affectivity (NA) is largely defined by sadness, fear, anger, frustration, and poor soothability. Surgency (SU) is mostly described by high activity, impulsivity, and sociability, and a low level of shyness. From the perspective of scholastic success, the least questionable is EC. Studies have highlighted its predominantly positive interface with a broad range of academic performance variables [33,34,35,36]. However, there were contradictory findings too. For instance, no relationship was found between EC and reading or mathematical achievements among preschoolers [37]. The direction of interface between NA and academic achievement was also found to be sensitive to multiple aspects. Negative associations of NA with school readiness were reported for preschool-age children [36,38]; for elementary school pupils, only anger—not sadness—produced an inverse relationship with achievement [39]. Teacher-rated NA of adolescents was associated with higher math grades, while self-rated NA had no link with them [40]. Studies reported two-fold associations of SU with school achievements, for instance, positive links with pre-academic skills [38] and reading achievement in the first grade [41], together with zero correlations with reading skills among school-age children [42].

Thus, there is a significant research body with rather inconsistent findings on the interface between a child’s temperament and school achievement. Therefore, there is a need to systematize the existing evidence-based findings to understand the central tendency of the relationship between temperament and academic achievement. We found extensive theoretical and empirical support for several factors, considered as moderators, for this relationship. These were as follows: educational level and transitional status, low socio-economic status (SES), and the sources of report about temperament and academic achievement.

### 1.2. Moderators

#### 1.2.1. Educational Level and Transitional Status

Children progressively climb three educational levels—pre-primary, primary, and secondary—defined by a change in structure of learning environment. There is some evidence that the magnitude of the relationship between temperament and achievement varies depending on educational level. Implications from Maziade et al.’s [29] study highlighted different patterns of this interface at age 7, compared to age 12. That is, more significant relationships were recorded at the older age; moreover, negative correlation was found between persistence and achievement at age 7 and positive correlation at age 12. In contrast, Al-Hendawi [43] reported decreasing tendencies of associations between temperament and achievement from childhood to adolescence. This finding is theoretically supported by Chess and Thomas [44], who suggested that the structure and even the nature of temperament is subjected to the growing influence of environmental forces throughout the school years.

During schooling years, pupils undergo two major transitions—from pre-primary to primary level and from primary to secondary level. Transitioning to an advanced educational setting is relatively short-term but has a very turbulent pace. Temperament operates on a full scale at the points of transition from one educational level to another. This has been the focus of many studies, such as those on the transition from preschool to primary school [45,46], primary to elementary school [34,47], and elementary to high school [48,49,50]. Several studies highlighted that adaptation to a new educational level is associated with the fall of academic grades [51,52]. The threat of deviation from an established academic pathway is especially evident at the transition to secondary school [50,53] when the pupil has to navigate the demands of multiple subject-teachers.

#### 1.2.2. Socio-Economic Status

A child’s temperament ties with academic achievement may vary by their family socio-economic status. Numerous studies have documented the unfavorable contribution of low SES to the links between children’s temperament and their academic success.

The NYLS—the first systematic study of temperament—suggested the importance of family SES for the child’s learning outcomes [12]. Currently, the impact of SES has been explored most extensively among pre-primary and primary school children. Existing empirical evidence suggested that EC is linked with the entire range of variables of academic success [33,35,54,55]. For instance, Razza et al. [56] found that the lack of impulsivity at age 5 was a strong predictor of emerging math and literacy skills at age 7 in the poorest, but not in the nearly poor children’s group. This tendency was not confirmed in the extended longitudinal study covering the period of elementary school—early attentional regulation predicted school achievement across both levels of poverty [45]. The authors tentatively explained this finding by the increased exposure to testing situations during primary school years, unlike the rare experiences in demand-eliciting situations in the families of low SES during early childhood. Similarly, McClelland et al. [57] found that pupils from low-income families, compared to those from middle-income families, enter the primary school with lower abilities to regulate their attention and behavior, which influence later under-performance at school. Other existing connections suggested that family SES was weakly related to their children’s EC [54] and NA [58]; in both cases, the relationship was more significant for literacy but not math skills.

Few studies have longitudinally examined the interaction of SES, temperament, and achievement during subsequent school years. It was affirmed that strong EC and low NA and SU buffered against learning difficulties that stemmed from low socio-economic background in the span from primary to secondary school [50]. Another longitudinal study derived the conclusion that temperament was also a significant predictor of achievement, at the end of school years, even after controlling SES [59]. Thus, from a longitudinal perspective, certain children’s temperament categories can counterbalance the risk posed by low family SES.

#### 1.2.3. Sources of Report on Temperament and Achievement

The current understanding on what a child’s temperament means for their achievement was accumulated predominantly using teachers’ and parents’ reports. Day-to-day interactions with children enable adults to assess their more pervasive, consistent traits. However, teachers and parents have disparate views of children’s individual differences [49,60]. While both value adaptability and learning-related persistence [61], they exhibit distinct opinions on a child’s emotionality and regulation [62,63], particularly on negative emotionality [64]. The NYLS findings suggested that parents could aggravate a child’s negative mood and adaptational troubles if they had rigid parenting standards and high expectations for school achievement [44]. In the school context, a teachers’ impression about students interfered with the grading of their performance [42,65]; additionally, these impressions were assumed to explain why children’s temperament was related to their achievement [40].

There are various sources of report on school achievement as well. It is commonly evaluated by the teacher-assigned grades (e.g., grade point average (GPA), rating scales) and by standardized testing. GPA is considered a more valid indicator of a student’s achievement because it generalizes the quality of many assignments over time [66], and it predicts students’ future academic achievement more accurately compared to other assessment methods [67]. It is also considered to be more sensitive to the individual differences of children [9,68] and to the subjectivity of teachers’ opinions [43]. Compared to GPA, standardized testing is assumed to be a more objective and a useful measure of students’ achievement [43,69]; however, its ability to adequately capture acquired knowledge and skills is limited [70,71]. Studies have shown that certain individual characteristics of children are more sensitive to a particular assessment method of achievement [72,73]. Therefore, both the teacher-assigned grades and standardized testing reflect different aspects of academic performance.

### 1.3. Present Study

Our decision to conduct a meta-analysis on the relationship between children’s temperament and their academic achievement was guided by the necessity for clear implications from previous studies [57]. Excluding a few reviews [9,43,74], there has been no meta-analysis on this issue. Meanwhile, some other non-cognitive correlates of academic outcomes—physical activity [75], creativity [76], personality [77,78], subjective well-being [79], self-concept [80], early life non-cognitive skills [2], and so on—have already been meta-analyzed.

Currently, a vast majority of empirical data on pupils’ temperament has been based on Rothbart’s model [31,32,81]. This model groups inherent psycho-biological characteristics into three dimensions (EC, NA, and SU), and captures the full range of child behaviors across all educational levels. It particularly emphasizes on children’s regulatory function, which is very important within the school context. There were also studies based on other theoretical approaches (the interactional and the criterial). We included them into our meta-analysis as well. In this case, temperament categories were considered as semantic equivalents of EC, NA, and SU, following the grouping by Else-Quest et al. [82] (p. 57).

Thus, our focus lay on the relationship of EC, NA, and SU with the overall, math, and reading achievement of children. To generalize the accumulated data on these links, we set two goals: first, to investigate the effect size between them; second, to examine the impact of potential moderators on the aforementioned relationship. Therefore, we expected to clarify the magnitude of the mainly positive and tentatively negative effects of EC and NA, respectively, on the school performance, with no clarity about the direction of the SU contribution. Those factors whose influence on the temperament–achievement relationship was already affirmed in a majority of existing studies, were used to tackle the plausible heterogeneity of studies. We believe that this meta-analysis will help to contextualize and specify the relationship between pupils’ temperament and their academic achievement.

## 2. Materials and Methods

This meta-analysis adhered to the Preferred Reporting Items for Systematic Reviews and Meta-Analyses (PRISMA) guidelines [83]. The protocol for this meta-analysis was not registered.

### 2.1. Eligibility Criteria

To be included in the analysis, a study had to meet the following criteria: (1) an original empirical research on the relationship between temperament and academic achievement; (2) conducted with school-age children; (3) reliable instruments and/or procedures; 4) only one empirical report was taken if the same sample results were presented in several sources.

### 2.2. Information Sources and Search Strategy

The literature search was conducted in December 2019 without limiting the search date. Three strategies were used. First, we searched for the studies in the following databases: Web of Science (Clarivate Analytics), ScienceDirect, and the package of EBSCO Publishing Databases (including PsycINFO, PsycARTICLES, ERIC, MEDLINE, SocINDEX, Teacher Reference Center, and OpenDissertations). We combined the term “temperament” with terms such as “academic achievement”, “academic performance”, “grade point average”, and “GPA” using the Boolean operators AND and OR. We searched the combinations of terms in titles, abstracts, and/or subject terms, depending on the capabilities of the databases. Second, we looked for unpublished studies. For this, we disseminated information on our meta-analysis on the Temperament Consortium website (https://www.b-di.com, accessed on 9 November 2019), which unites temperament professionals around the world. We also contacted the Australian Temperament Project (https://www.melbournechildrens.com, accessed on 9 November 2019) with a request to share unpublished research data on temperament and academic achievement. Third, we conducted a backward literature search in the references of studies already included.

### 2.3. Data Collection Process and Coding

#### 2.3.1. Effect Measure and Main Variables

The effect size was a bivariate correlation coefficient between temperament and academic achievement. All temperament variables presented in the studies were assigned in terms of meaning to one of the three dimensions—EC, NA, or SU. The teacher assessments and the results of the standardized achievement tests were considered as academic achievement indicators. These variables were assigned to mathematics, reading, and overall (i.e., several curriculum subjects). When a few correlation coefficients between temperament and achievement were presented in the same study, they were averaged, transforming each coefficient into Fisher’s *z* and converting back to *r* after averaging [84,85]. Averaging was necessary when the study reported correlations between (a) several indicators of the same temperament dimension and academic achievement, (b) temperament and several indicators of academic achievement, (c) temperament and academic achievement in the same sample at different times (longitudinal studies), and (d) temperament and academic achievement from more than one informant (e.g., teachers and parents). Averaging was not applied when correlation coefficients were provided on how a broad dimension (e.g., EC) and its components (e.g., inhibitory control, attention focusing) were linked with the other broad domain (e.g., reading) and its components (e.g., letter–word identification, reading fluency). To further the analysis, preference was given to the broad variables. Thus, only one correlation coefficient was taken from one sample, showing a relationship between the same dimension of temperament and the same domain of academic achievement.

#### 2.3.2. Other Variables

Selected studies included children from different educational levels. Some studies covered several educational levels at the same time. The educational level was coded according to when academic achievement was assessed (*pre-primary* = 1, *primary* = 2, *secondary* = 3). If the educational level was not specified in the study, we relied on country-specific information [86] according to the age and/or grade of the participants. We did not include studies that combined children from two educational levels, namely, both pre-primary and primary [87,88,89,90,91] and both primary and secondary [30,92,93,94,95]. We classified these studies into *transition* (= 1) or *non-transition* (= 0).

We categorized all the studies by SES risk (*non-risk* = 0, *risk* = 1). If the authors did not classify their sample by socio-economic origin, we assigned a sample to the risk/non-risk group based on the sample description. For example, if most participants were indicated as receiving free meals, we identified such a sample as SES risk; if most participants belonged to the middle class, we assigned such a sample to the non-risk group.

The sources of information about temperament were *parents* (= 1), *teachers* (= 2), or *self-report* (= 3). When information was obtained from several sources (e.g., both parents and teachers), the source was coded as *multiple* (= 4). There was only one study [34] in which temperament was assessed using laboratory procedure; so, we did not include it in this moderator analysis. Sources of information on academic achievement were *teacher assessments* (= 1) or *standardized achievement test scores* (= 2). When information was obtained from several sources (e.g., both grades and standardized tests), the source was coded as *multiple* (= 3).

Prior to coding, a coding protocol was developed. Both authors tested it independently on 20% of randomly selected studies. After discussing the issues raised, the coding system was improved, and the rest of the study was coded by one of the authors. Data analysis was initiated only after full consensus was reached.

### 2.4. Synthesis Methods

To answer questions about the central tendency of relation between temperament and academic achievement, the average effect size was calculated using the Fisher *r*-to-*z* transformed correlation coefficient as the outcome measure. A random-effects model was fitted to the data [96,97,98]. The studies were weighted using their sampling variance and the estimated amount of heterogeneity [99]. The relationships between the three dimensions of temperament (EC, NA, and SU) and the three domains of academic achievement (overall, math, and reading) were calculated separately. The benchmark values of 0.10, 0.20, and 0.30 for small, medium, and large effect size, respectively, were chosen [100,101]. The amount of effect size heterogeneity (τ^2^) was estimated using the restricted maximum-likelihood estimator [102] also taking the *Q*-test for heterogeneity [103] and the *I*^2^ statistic [104]. The *Q*-test revealed the fact of heterogeneity (when *p* < 0.05) [105] while *I*^2^ values of 25%, 50%, and 75% meant low, medium, and high inconsistency, respectively [104].

We also inspected the presence of outliers and/or influential studies. Studies with a studentized residual larger than the 100 × (1 − 0.05/(2 × *k*))th percentile of a standard normal distribution were considered potential outliers. Studies with a Cook’s distance larger than the median plus six times the interquartile range of the Cook’s distances were influential [106]. The rank correlation test [107] and the regression test [108], using the standard error of the observed outcomes as predictor, were used to check for funnel plot asymmetry. In both cases, statistical significance (*p* < 0.05) indicated evidence of publication bias.

To explain the heterogeneity across studies, we conducted the analysis of each moderator. A mixed-effects model was fitted to the data using the restricted maximum-likelihood estimator [97,109]. All moderators were categorical, and the moderator was considered significant, if the effect size differed statistically significantly (*p* < 0.05) across groups [110,111].

The statistical analysis was carried out using the metafor package (version 2.1.0) [99], the multcomp package (version 1.4-13) [112], and the dmetar package (version 0.0.9000) [113] in R (version 3.6.2) [114].

## 3. Results

### 3.1. Study Selection

**Figure 1:**
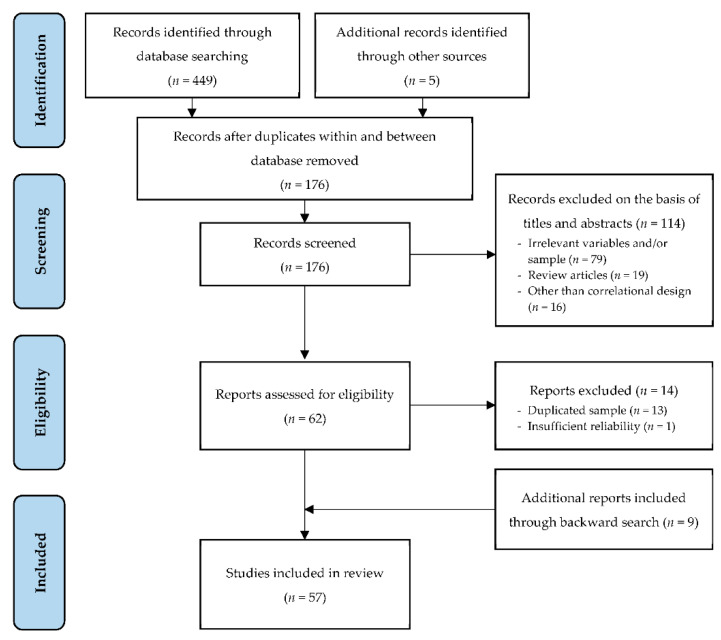
Flow diagram.

The results of study search and selection process are presented in a flow diagram (Figure 1) and described below.

The initial search yielded 454 records. Identical records within and between database were excluded before screening and 176 records remained. We reviewed the titles and abstracts of these records and those that did not meet the first two eligibility criteria (e.g., lack of temperament and/or academic achievement variables, study sample age beyond our set limits, review articles, other than correlational design) were excluded from further analysis. We thoroughly assessed remained 62 reports and excluded 14 that did not meet the third and fourth eligibility criteria. Namely, several studies presented results of the same sample, such as publications from the Study of Early Child Care and Youth Development at the National Institute of Child Health and Human Development or the Finnish Study of Temperament and School. To avoid duplication of the sample across many publications from the same project, we selected an article that covered more relevant variables and/or a larger sample. One more reason for exclusion was the low reliability of temperament measures (e.g., Cronbach’s value of the scales < 0.60). We carefully reviewed the bibliographies of the selected reports and such backward search added nine more studies. The result of the study selection included 57 original studies on the relationship between temperament and academic achievement.

### 3.2. Description of Studies

Fifty-seven original studies were selected for the meta-analysis: 48 published articles (84.2%), 7 doctoral dissertations (12.3%), and 2 with unpublished research data (3.5%). These studies included research on the relationship between temperament and academic achievement in 12 countries from 1985 to 2019. The 47 samples represented one level of education: 6 pre-primaries (10.5%), 28 primaries (49.1%), and 13 secondaries (22.8%), while 10 samples (17.5%) were transitional (i.e., from two educational levels). Forty-two samples (73.7%) were selected from diverse SES backgrounds, and fifteen samples (26.3%) were assigned to the SES risk group. In 18 samples, the sources of information on temperament were the parents (31.6%), in 17 samples—the teachers (29.8%), 9 samples—self-report (15.8%), 12 samples—multiple sources (21.1%), and in one study, the temperament was assessed by a laboratory procedure (1.8%). In 25 samples (43.9%), the information on achievement was provided by the teachers, in 19 samples standardized tests (33.3%) were used, and in 13 samples the achievement data relied on multiple sources (22.8%). Detailed descriptive information on each study is provided in Table 1.

### 3.3. Average Effect Size

Nine average effect sizes, expressed in Fisher *r*-to-*z* transformed correlation coefficients, were obtained to describe the relationship between the three dimensions of temperament (EC, NA, and SU) and the three domains of academic achievement (overall, math, and reading) (Table 2). Neither the rank correlation (Kendal’s tau), nor the regression test (Egger’s test), indicated any funnel plot asymmetry (*p* > 0.05). Below, the results are presented in a more detailed and sequential manner, according to the relationship of each of the three dimensions of temperament with the three domains of academic achievement. A complete list of studies with individual effect sizes can be found in Appendix A.

Correlations between EC and academic achievement ranged from −0.10 to 0.78 (overall), −0.12 to 0.55 (math), and −0.08 to 0.63 (reading). Most of the estimates (96–98%) were positive. There was no indication of outliers across all three models; however, one study appeared to be overly influential for the EC and overall achievement model [116].

Similarly, correlations of NA with academic achievement ranged from −0.37 to 0.09 (overall), −0.45 to 0.25 (math), −0.39 to 0.00 (reading), with most estimates (90–95%) being negative. Moreover, no indication of outliers was present for these models, but one study [130] was considered as overly influential in the NA and overall achievement model. In addition, one study [119] had a relatively larger weight compared to the rest of the studies in the NA and math achievement model.

Correlations between SU and overall achievement ranged from −0.30 to 0.44, and one study [116] may be a potential outlier as well as overly influential for this model. Relationships between SU and math achievement ranged from −0.30 to 13, and this model had no outliers. However, one study [123] could be considered as overly influential. Lastly, correlation coefficients between SU and reading achievement ranged from −0.25 to 0.44. One study [93] may be a potential outlier in this model; furthermore, two studies [87,120] could be overly influential. In all three models, slightly more estimates (52–60%) were negative.

In sum, the highest average effect sizes—from medium to large—were obtained between the EC and overall, math, and reading achievement. Small negative average effect sizes were found between the NA and all the three academic achievement variables while effect sizes between academic achievement and SU was received close to zero and were statistically non-significant. Analysis of heterogeneity showed that all the average effect sizes were significantly heterogenous with low to high inconsistency. This meant that average effect sizes could be affected by potential moderators.

### 3.4. Analysis of Moderators

A moderator analysis was performed to verify whether the average effect size between temperament and achievement varied between groups by educational level, transition status, SES risk, and source of information on temperament and achievement (Table 3).

Analyses revealed that educational level (pre-primary, primary, secondary), transition status (transition, non-transition), and SES risk (risk, no risk) were not statistically significant moderators. In all these cases, *Q* value was low and not significant as well as the categories of these moderators did not differ statistically significantly from their reference categories (intercepts). That is, the temperament–achievement relationships were similar—statistically significantly or not—between different categories of educational level, transition status, and SES risk.

Conversely, sources of information on both temperament (parents, teachers, self-report, multiple) and academic achievement (teacher assessments, test scores, multiple) were statistically significant moderators. The results showed statistically significant differences among effect sizes of EC in relation to math achievement (*Q*(3) = 8.86, *p* < 0.05); effect size was significantly higher when information was provided by teachers compared to parents (intercept) (*p* < 0.01). Significant differences were also observed in effect sizes between NA and math achievement (*Q*(3) = 11.92, *p* < 0.01) as well as reading achievement (*Q*(3) = 14.58, *p* < 0.01). Specifically, when parents (intercept) reported on the child’s temperament, the effect size between NA and math was statistically significantly lower, compared to reports from teachers (*p* < 0.05), self (*p* < 0.05), and multiple sources (*p* < 0.05). A very similar result was found for the effect size between NA and reading; a statistically significantly lower effect size was found when the source of information was parents (intercept) compared to when the sources were teachers (*p* < 0.001) and multiple (*p* < 0.05). The results revealed statistically significant differences among effect sizes of EC and overall achievement depending on the source of the information on academic achievement (*Q*(2) = 12.48, *p* < 0.01). Effect size was significantly higher when achievement was assessed by the teachers (intercept) compared to test scores (*p* < 0.001).

In sum, neither the education level nor the transition status and the SES risk had a statistically significant influence on the relationship between temperament and academic achievement. Only the source of information (on temperament and academic achievement) appeared to have a statistically significant influence on relation between temperament and academic achievement variables.

## 4. Discussion

To the best of our knowledge, our meta-analysis was the first attempt to synthesize the empirical findings on the relationship between three temperament dimensions and academic achievement in school children. It appeared that the three temperament dimensions—EC, NA, and SU—were positively, negatively, and unclearly, linked with school achievement, respectively. EC and NA interacted with achievement in a very narrow range of significant effect size, regardless of the academic subject. In other words, the average of the relationships between EC and math was 0.24, between EC and reading was 0.25, between NA and math was −0.13, and between NA and reading was −0.14. The very close effect sizes in each dimension, together with a lack of outliers, suggested that the existing research confirmed a definite contribution of EC and NA to achievement. Each result seemed to be a part of a larger study on this particular relationship, despite differences in sample size or educational level.

Regarding positive EC links with children’s achievement, this was expected. Compared with findings from the very few published secondary analyses, our conclusion specified this relationship and challenged the notion that it was dependent on the nature of the academic subject. Specifically, a previous meta-analysis on a particular facet of EC—inhibitory control—in preschoolers reported it to be stronger associated with math-related skills, compared with literacy-related ones [4]. Another meta-analytic investigation that also dealt with children’s individual differences in relation to their achievement [78] suggested that in primary education, the strongest predictor of achievement was a child’s conscientiousness, which was believed to develop from the child’s EC [145].

There was no documented evidence summarizing the relationship of NA and achievement from individual studies; however, there are some findings on NA-related characteristics. For instance, negative arousal was found to be inversely linked to academic achievement [146]. Extensive research highlighted that negative emotionality hindered a child’s capacity to develop the skills needed for success in school [147,148,149]. Additionally, there were sporadic findings of no associations between NA and academic outcomes [150] and split opinions by caregivers and teachers on this issue [142]. Thus, we claim our testimony of definite inverse links of NA with academic achievement to be an incremental finding of our meta-analysis.

The evidence on SU collected so far appeared to be so conflicted that the overall size of the relationship between SU and scholastic achievement yielded an insignificant outcome. The effects were distributed over a very wide range from −0.30 to 0.44, with several outliers and influential studies. Even from a theoretical perspective, the multifaceted composition of SU could be related to a mixed contribution to academic achievement. On the one hand, it comprises an activity level. Some studies claimed its positive link to academic outcomes [151,152] or at least to math grades [11]. On the other hand, SU also encompasses impulsivity, which is typically negatively linked to achievements [45,153]. Therefore, it is possible that one facet of SU “neutralized” the other during aggregation of primary findings.

The moderator analysis helped to specify the discovered central tendencies within the educational context. Educational level and transition status were not statistically significant as moderators, thereby supporting the testimony of other authors [154,155] that the temperament–achievement connection is stable over the school years. Additionally, SES was also not significant moderator. Its hypothesized moderator capacity was possibly weakened by the inconsistency of SES measurement among different studies: (1) based on the family income [56], (2) a composite indicator for poor districts in a particular state [87], or 3) an averaged index of socioeconomic risk [50].

Sources of information on temperament and achievement appeared to influence the relationship in question. It was affirmed that children’s EC and NA were more strongly related to achievement, when temperament was reported by teachers than parents. Additionally, the relationship between EC and performance was stronger in the case of teacher-assigned grades than standardized testing. This meta-analytic message suggests that teachers’ perceptions of child temperament could affect their pedagogical decisions, which was consistent with other reports. For instance, it was found [156] that teachers tended to overestimate the achievement level of their class and were barely accurate in the assessment of negative emotions of the students. Deater-Deckard et al. [42] provided evidence that perceived polarities of pupils’ temperament were related to the mismatch between the assigned grades and children’s abilities. On the other hand, there are reports [157] supporting the accuracy of teachers’ assessments.

### 4.1. Implications

Our aggregated results clearly indicated the definite advantage of EC and the obvious disadvantage of NA for achievement outcomes. A child with high EC learns in a more autonomous way and is more self-reliant in the classroom with a minimum need for monitoring. A child with high NA has a different approach to school assignments and a very limited capacity to overcome their mood extremes. Frustration, irritability, and anger could jeopardize the realization of their full learning potential. Thus, children with high NA need to be taught to recognize and cope with these primary reactions, to embrace their strengths, rather than persisting in negative states.

These findings require a mindful reflection. Teacher-assigned grades seemed to have an objective and subjective component—the evaluation of accumulated knowledge and the assessment of temperament, respectively. The contribution of a non-cognitive factor to academic achievement poses the threat of educational labelling, especially for negative emotionality. From this perspective, the very concept of possibly more or less “teachable” children or “ideal” students could be questionable. Every child is teachable in their own way and pace.

Moreover, ignoring a child’s temperament may lead to their frustration and a defensive stance towards learning [158] and cause misinterpretation of feelings such as frustration as disobedience [65,159]. Several intervention programs [160,161,162] and scientific projects [64,163] have already demonstrated the effectiveness of tailoring education to pupils’ temperament. Their incremental value can be seen through the various benefits for parents, children and teachers as follows: (1) It enabled parents to understand the basics of child’s tentatively differentiated behavior in home and school. (2) It allowed pupils to identify their unique learning style, and implement their strengths and move beyond their limitations. (3) It paved the way for teachers to engage in evidence-based teaching, by being a more effective teacher, who is sensitive to individual differences, rather than working harder. (4) Additionally, it enhances teachers’ awareness of the effect of their own temperament on their professional decisions and expectations.

These benefits may serve as guidelines for the development of educational policies. Temperamental attributes lay the groundwork for personalized learning and provide a framework to identify their own individuality in the classroom. The growing diversity in education requires constant renewal and review of effects of the patterns of temperamental characteristics in transforming the teachings into learnings. These issues should become an integral part of teachers’ trainings.

### 4.2. Limitations and Guidelines for Future Studies

There are certain constraints regarding the generalizability of our results. A different conceptual framework of temperament or choice of moderators would possibly yield different and challenging results. The adoption of a tri-partite constellation of temperament, which generated most of the existing research, required us to classify the attributes into three broad groups. It confined our sensitivity to a genuine understanding of authors. We relied on the capability of meta-analyses to provide a synthesized solution, i.e., a broader picture, by looking “at the forest instead of trees”. Certain distinct characteristics in one particular dimension could act contradictorily within educational settings. Therefore, a meta-analysis on the broad categories of temperament combined with their narrower attributes would be useful in the future.

In most cases, the moderator analysis was conducted with an asymmetrical number of studies in the comparable groups. The synthesized picture of the research fields documented the frequency for the investigation of some less common samples. Although there are no strict guidelines on the number of groups to be compared within a meta-analysis, the results of such a comparison should be interpreted with some caution. For instance, this meta-analysis was limited due to teachers’ and parents’ reports dominance over self-reports and laboratory assessments of temperament; furthermore, primary educational level was investigated more than the secondary level. According to our observation, contemporary research involves a wider range of data sources and information from a variety of contexts. Thus, future original studies can be expected to include a bigger variety of data sources, a wider age-range of participants, and assessment of temperament in real-life situations.

## 5. Conclusions

The main findings of this meta-analysis confirmed the various contributions of distinct temperament dimensions—the affirmative, unfavorable, and indefinite effect of EC, NA, and SU, respectively—to a child’s academic performance. Contrary to expectations, some of the selected moderators—educational level, transition status, and family’s SES—did not reduce the heterogeneity of studies on the link between achievement and particular dimensions of temperament. Meanwhile, the sources of the report appeared to be relevant. Specifically, the relationship between children’s temperament and their achievement was significantly stronger when the teachers provided data on child’s temperament and when teachers assigned the grades.

## Figures and Tables

**Table 1:** Descriptive information of studies included in the meta-analysis.

Author(s), Year	Type of Source	Country ^1^	Outcome		Educational Level/Transition	SES Risk	Information on	
			Temperament	Achievement			Temperament	Achievement
Al-Hendavi, 2010 [115]	Dissertation	US	EC, NA, SU	O	Primary	Yes	Teachers	Teachers
Blair and Razza, 2007 [33]	Article	US	EC	O, M, R	Pre-primary	Yes	Teachers	Test
Bruni et al., 2006 [116]	Article	IT	EC, NA, SU	O	Primary	No	Teachers	Teachers
Bryce et al., 2018 [117]	Article	US	EC, NA, SU	O	Pre-primary	Yes	Teachers	Test
Checa and Abundis-Gutierrez, 2017 [92]	Article	ES	EC, NA	O	Transition	No	Parents	Teachers
Checa et al., 2008 [118]	Article	ES	EC, NA, SU	O, M	Secondary	No	Multiple	Teachers
Chen et al., 2015 [54]	Article	US	EC	O, M, R	Primary	No	Multiple	Test
Chong et al., 2019 [119]	Unpublished	AU	EC, NA, SU	O, M, R	Primary	No	Parents	Teachers
Colom et al., 2007 [120]	Article	ES	NA, SU	O, M, R	Secondary	No	Self	Teachers
Dindo et al., 2017 [121]	Article	US	EC	O, M, R	Secondary	No	Multiple	Test
Fox et al., 2001–2010 [122]	Unpublished	US	EC, NA, SU	O, M, R	Primary	No	Parents	Test
Gaias et al., 2016 [123]	Article	US	EC, NA, SU	O, M, R	Primary	No	Teachers	Test
Galian et al., 2018 [124]	Article	ES	EC	R	Primary	No	Parents	Teachers
Gullesserian, 2009 [125]	Dissertation	US	EC, NA, SU	O, M, R	Secondary	No	Parents	Multiple
Gumora and Arsenio, 2002 [126]	Article	US	NA, SU	O	Secondary	No	Multiple	Multiple
Han et al., 2017 [127]	Article	US	EC, NA, SU	R	Pre-primary	Yes	Parents	Test
Hegvik, 1985 [93]	Dissertation	US	EC, NA, SU	O, M, R	Transition	No	Parents	Teachers
Hernandez, 2002 [128]	Dissertation	US	EC, NA, SU	O, M, R	Primary	No	Teachers	Multiple
Hirvonen et al., 2013 [129]	Article	FI	EC, NA, SU	O, M, R	Primary	No	Teachers	Test
Hirvonen et al., 2019 [130]	Article	FI	EC, NA	O	Secondary	No	Parents	Teachers
Hsieh, 1998 [131]	Dissertation	TW	NA, SU	O	Primary	No	Multiple	Teachers
Huang and Yeh, 2019 [47]	Article	TW	EC, NA	R	Primary	No	Self	Multiple
Hughes and Coplan, 2010 [94]	Article	CA	SU	O, M, R	Transition	No	Self	Multiple
Iyer et al., 2010 [132]	Article	US	EC	O	Primary	Yes	Teachers	Teachers
Jeronimus et al., 2015 [133]	Article	NL	NA	O	Secondary	No	Parents	Teachers
Johns et al., 2019 [58]	Article	US	EC, NA	O, M, R	Pre-primary	No	Multiple	Test
Kornienko et al., 2018 [134]	Article	RU	EC	O	Primary	No	Parents	Teachers
Kwon et al., 2018 [62]	Article	US	NA	R	Primary	Yes	Teachers	Test
Liew et al., 2008 [34]	Article	US	EC	O, M, R	Primary	No	Laboratory	Test
Liu et al., 2018 [37]	Article	US	EC, NA, SU	O, M, R	Primary	No	Parents	Test
Marcynyszyn, 2006 [135]	Dissertation	US	EC	O, M, R	Primary	Yes	Parents	Teachers
Martin et al., 1988, Study 1 [11]	Article	US	EC, NA, SU	O, M, R	Primary	No	Teachers	Multiple
Martin et al., 1988, Study 2 [11]	Article	US	EC, NA, SU	O, M, R	Primary	No	Teachers	Multiple
Martin et al., 1988, Study 3 [11]	Article	US	EC, NA, SU	O, M, R	Primary	Yes	Teachers	Multiple
Martin and Holbrook, 1985 [28]	Article	US	EC, NA, SU	O, M, R	Primary	Yes	Teachers	Multiple
Miller, 1999 [95]	Dissertation	US	EC, NA, SU	O, M, R	Transition	Yes	Teachers	Multiple
Moreira et al., 2012 [136]	Article	PT	EC, SU	O	Secondary	Yes	Self	Teachers
Morris et al., 2013 [137]	Article	US	EC	O, M, R	Pre-primary	Yes	Teachers	Teachers
Mullola et al., 2014 [60]	Article	FI	EC, NA, SU	M, R	Secondary	No	Multiple	Teachers
Oades-Sese et al., 2011 [87]	Article	US	NA, SU	O, M, R	Transition	Yes	Teachers	Test
Oliver et al., 2007 [138]	Article	US	EC	O, M, R	Secondary	No	Multiple	Teachers
Ooi et al., 2017 [88]	Article	CA	NA, SU	O	Transition	No	Parents	Teachers
Palisin, 1986 [139]	Article	US	EC, NA, SU	O	Pre-primary	No	Parents	Test
Raymo et al., 2019 [140]	Article	US	EC	O	Secondary	No	Self	Teachers
Razza et al., 2012 [45]	Article	US	EC	O, M, R	Primary	Yes	Self	Test
Sanchez-Perez et al., 2018 [35]	Article	ES	EC	O, M, R	Primary	No	Parents	Multiple
Scrimin et al., 2019 [48]	Article	IT	NA, SU	O	Secondary	No	Self	Teachers
Studer-Luethi et al., 2016 [141]	Article	CH	EC	O, M, R	Primary	No	Multiple	Test
Swanson et al., 2014 [89]	Article	US	EC	M	Transition	No	Parents	Test
Talwar et al., 1989 [30]	Article	US	NA, SU	O	Transition	No	Self	Multiple
Valiente et al., 2013 [49]	Article	US	EC, SU	O	Primary	No	Multiple	Teachers
Valiente et al., 2014 [90]	Article	US	EC	O	Transition	No	Multiple	Multiple
Wang et al., 2017, Study 1 [50]	Article	US	EC, NA, SU	O, M, R	Primary	No	Parents	Test
Wang et al., 2017, Study 2 [50]	Article	US	EC, NA, SU	O, M, R	Primary	Yes	Parents	Test
Zhang et al., 2017 [91]	Article	US	SU	O	Transition	No	Teachers	Teachers
Zhou et al. 2010 [142]	Article	CN	EC, NA	O	Primary	No	Multiple	Teachers
Zorza et al. 2019 [143]	Article	ES	EC	O	Secondary	No	Self	Teachers

^1^ Country abbreviation are given in accordance with the International Organization for Standardization [144]. EC = effortful control; NA = negative affectivity; SU = surgency; O = overall academic achievement; M = math achievement; R = reading achievement.

**Table 2:** Results of average effect size and test of heterogeneity.

Variables	*k*	*N*	ES*_r_*	95% CI	*SE*	*z*	Test of Heterogeneity		
							τ^2^	*Q*(*df*)	*I* ^2^
Effortful control									
×Overall	41	14,126	0.31	[0.26, 0.37]	0.03	11.14 ***	0.03	371.82 (40) ***	88.77
×Math	28	10,852	0.24	[0.19, 0.30]	0.03	8.96 ***	0.01	145.73 (27) ***	82.34
×Reading	30	11,165	0.25	[0.19, 0.30]	0.03	9.09 ***	0.01	154.04 (29) ***	82.78
Negative affectivity									
×Overall	32	10,062	−0.13	[−0.17, −0.10]	0.02	−6.98 ***	0.01	72.01 (31) ***	60.44
×Math	20	6538	−0.13	[−0.17, −0.09]	0.02	−6.85 ***	0.00	31.54 (19) *	33.66
×Reading	22	6817	−0.14	[−0.18, −0.09]	0.02	−5.79 ***	0.01	43.69 (21) **	59.24
Surgency									
×Overall	31	7632	−0.00	[−0.06, 0.06]	0.03	−0.03	0.02	128.60 (30) ***	83.06
×Math	19	6333	−0.05	[−0.10, 0.00]	0.03	−1.80	0.01	42.01 (18) **	65.36
×Reading	20	6388	−0.04	[−0.08, 0.01]	0.02	−1.54	0.01	43.53 (19) ***	54.91

*k* = number of studies; *N* = sample size; ES*_r_* = average effect size; CI = confidence interval; *SE* = standard error; *z* = test for significance of ES*r*; τ^2^ = estimated amount of total heterogeneity; *Q* = test for heterogeneity; *df* = degrees of freedom; *I*^2^ = total variability (%). * *p* < 0.05. ** *p* < 0.01. *** *p* < 0.001.

**Table 3:** Moderator analysis: educational level, transition status, SES risk, and source of information on temperament and academic achievement.

Variables	Overall Achievement			Mathematics			Reading		
	*k*	ES*r*	95% CI	*k*	ES*r*	95% CI	*k*	ES*r*	95% CI
Educational level × EC	*Q*(2) = 2.34			*Q*(2) = 1.46			*Q*(2) = 0.27		
Pre-primary ^1^	5	0.27 ***	[0.11, 0.42]	3	0.33 ***	[0.17, 0.49]	4	0.22 **	[0.09, 0.36]
Primary	24	0.29 ***	[0.22, 0.22]	18	0.23 ***	[0.17, 0.30]	20	0.24 ***	[0.18, 0.30]
Secondary	8	0.39 ***	[0.27, 27]	5	0.28 ***	[0.14, 0.42]	4	0.28 ***	[0.13, 0.43]
Educational level × NA	*Q*(2) = 1.27			*Q*(2) = 4.34			*Q*(2) = 1.75		
Pre-primary ^1^	3	−0.07	[−0.19, 0.06]	1	−0.16 **	[−0.28, −0.05]	2	−0.09	[−0.23, 0.05]
Primary	16	−0.13 ***	[−0.18, 0.07]	12	−0.10 ***	[−0.12, −0.07]	14	−0.13 ***	[−0.19, −0.07]
Secondary	7	−0.15 ***	[−0.23. 0.07]	4	−0.17 ***	[−0.23, −0.10]	3	−0.21 **	[−0.35, −0.08]
Educational level × SU	*Q*(2) = 0.22			*Q*(2) = 0.02			*Q*(2) = 0.45		
Pre-primary ^1^	2	−0.05	[−0.31, 0.20]	–	–	–	1	−0.02	[−0.19, 0.16]
Primary	16	−0.01	[−0.10, 0.07]	12	−0.03	[−0.09, 0.03]	12	−0.03	[−0.08, 0.03]
Secondary	6	0.02	[−0.14, 0.16]	4	−0.04	[−0.16, 0.07]	3	−0.07	[−0.18, 0.05]
Transition status × EC	*Q*(1) = 0.01			*Q*(1) = 0.66			*Q*(1) = 0.02		
Non-transition ^1^	37	0.31 ***	[0.25, 0.37]	26	0.25 ***	[0.19, 0.31]	28	0.24 ***	[0.19, 0.30]
Transition	4	0.32 ***	[0.14, 0.50]	2	0.17	[−0.03, 0.36]	2	0.26 *	[0.03, 0.49]
Transition status × NA	Q(1) = 1.07			Q(1) = 1.33			Q(1) = 0.00		
Non-transition ^1^	26	−0.13 ***	[−0.17, −0.08]	17	−0.12 ***	[−0.16, −0.09]	19	−0.14 ***	[−0.19, −0.09]
Transition	6	−0.18 ***	[−0.27, −0.09]	3	−0.20 **	[−0.33, −0.07]	3	−0.14	[−0.28, 0.01]
Transition status × SU	*Q*(1) = 0.24			*Q*(1) = 1.44			*Q*(1) = 0.00		
Non-transition ^1^	24	−0.01	[−0.08, 0.06]	16	−0.04	[−0.09, 0.02]	16	−0.04	[−0.09, 0.02]
Transition	7	0.03	[−0.11, 0.16]	3	−0.13	[−0.27, 0.01]	4	−0.04	[−0.16, 0.08]
SES risk × EC	*Q*(1) = 0.59			*Q*(1) = 0.68			*Q*(1) = 0.01		
Non-risk ^1^	29	0.33 ***	[0.26, 0.39]	20	0.23 ***	[0.17, 0.29]	21	0.25 ***	[0.18, 0.31]
Risk	12	0.28 ***	[0.18, 0.38]	8	0.28 ***	[0.18, 0.38]	9	0.24 ***	[0.15, 0.34]
SES risk × NA	*Q*(1) = 0.99			*Q*(1) = 0.22			*Q*(1) = 0.28		
Non-risk ^1^	25	−0.14 ***	[−0.19, −0.10]	15	−0.14 ***	[−0.18, −0.09]	15	−0.15 ***	[−0.20, −0.09]
Risk	7	−0.10 *	[−0.18, −0.02]	5	−0.12 **	[−0.19, −0.04]	7	−0.12 **	[−0.20, −0.04]
SES risk × SU	*Q*(1) = 2.39			*Q*(1) = 0.43			*Q*(1) = 0.18		
Non-risk^1^	23	0.03	[−0.04, 0.10]	14	−0.06	[−0.12, 0.00]	14	−0.03	[−0.09, 0.03]
Risk	8	−0.08	[−0.20, 0.04]	5	−0.02	[−0.13, 0.09]	6	−0.05	[−0.14, 0.03]
Temperament source × EC	*Q*(3) = 3.01			*Q*(3) = 8.86*			*Q*(3) = 5.05		
Parents ^1^	13	0.25 ***	[0.15, 0.34]	9	0.15 ***	[0.07, 0.23]	11	0.19 ***	[.11, 0.27]
Teachers	14	0.36 ***	[0.26, 0.46]	10	0.32 ***	[0.23, 0.41]	10	0.33 ***	[.23, 0.42]
Self	4	0.35 ***	[0.18, 0.51]	1	0.22 *	[0.01, 0.43]	2	0.22 *	[.04, 0.41]
Multiple	9	0.34 ***	[0.23, 0.46]	7	0.30 ***	[0.20, 0.40]	6	0.27 ***	[.17, 0.38]
Temperament source × NA	*Q*(3) = 3.17			*Q*(3) = 11.92 **			*Q*(3) = 14.58 **		
Parents ^1^	12	−0.10 ***	[−0.16, −0.05]	7	−0.09 ***	[−0.11, −0.06]	8	−0.07 **	[−0.11, −0.02]
Teachers	12	−0.16 ***	[−0.23, −0.09]	9	−0.17 ***	[−0.24, −0.11]	10	−0.20 ***	[−0.26, −0.14]
Self	3	−0.21 **	[−0.34, −0.08]	1	−0.27 **	[−0.44, −0.10]	2	−0.21 **	[−0.36, −0.06]
Multiple	5	−0.14 **	[−0.23, −0.04]	3	−0.16***	[−0.23, −0.10]	2	−0.18 ***	[−0.27, −0.09]
Temperament source × SU	*Q*(3) = 2.26			*Q*(3) = 4.02			*Q*(3) = 6.62		
Parents ^1^	9	0.03	[−0.09, 0.15]	6	−0.01	[−0.09, 0.07]	8	−0.00	[−0.06, 0.06]
Teachers	13	0.01	[−0.09, 0.11]	9	−0.08	[−0.16, 0.01]	9	−0.05	[−0.12, 0.02]
Self	5	−0.11	[−0.26, 0.05]	2	−0.17 *	[−0.34, −0.01]	2	−0.20 **	[−0.34, −0.05]
Multiple	4	0.04	[−0.14, 0.21]	2	−0.01	[−0.16, 0.14]	1	−0.00	[−0.15, 0.14]
Achievement source × EC	*Q*(2) = 12.48 **			*Q*(2) = 0.96			*Q*(2) = 1.87		
Subject grades ^1^	17	0.41 ***	[0.33, 0.48]	6	0.29 ***	[0.17, 0.41]	7	0.30 ***	[.18, 0.41]
Test scores	15	0.22 ***	[0.14, 0.30]	14	0.22 ***	[0.15, 0.29]	14	0.21 ***	[.14, 0.28]
Multiple	9	0.28 ***	[0.16, 0.39]	8	0.26 ***	[0.14, 0.38]	9	0.28 ***	[.16, 0.39]
Achievement source × NA	*Q*(2) = 2.41			*Q*(2) = 1.00			*Q*(2) = 1.22		
Subject grades ^1^	13	−0.14 ***	[−0.20, −0.09]	5	−0.17 ***	[−0.25, −0.09]	4	−0.18 ***	[−0.28, −0.08]
Test scores	10	−0.10 **	[−0.16, −0.04]	8	−0.12 ***	[−0.18, −0.06]	10	−0.12 ***	[−0.18, −0.05]
Multiple	9	−0.18 ***	[−0.27, −0.10]	7	−0.12 *	[−0.22, −0.03]	8	−0.14 **	[−0.24, −0.04]
Achievement source × SU	*Q*(2) = 1.96			*Q*(2) = 0.54			*Q*(2) = 1.01		
Subject grades ^1^	12	0.05	[−0.05, 0.15]	4	−0.02	[−0.13, 0.10]	4	0.01	[−0.09, 0.11]
Test scores	9	−0.05	[−0.16, 0.06]	7	−0.07	[−0.16, 0.02]	8	−0.05	[−0.12, 0.02]
Multiple	10	−0.02	[−0.14, 0.10]	8	−0.05	[−0.15, 0.06]	8	−0.05	[−0.15, 0.04]

^1^ The reference category (intercept). For convenience, real values of effect size and confidence interval are presented, whereas analysis for heterogeneity results are presented with intercept. Asterisks next to effect sizes indicate the statistical significance of the temperament–achievement relationship for each category of moderator. EC = effortful control; NA = negative affectivity; SU = surgency; *k* = number of studies; ES*r* = average effect size; CI = confidence interval; *Q* = test of moderators (omnibus). * *p* < 0.05. ** *p* < 0.01. *** *p* < 0.001.

## Appendix A

**Table A1:** List of studies with individual effect sizes: Effortful control and academic achievement.

Author(s), Year	Overall Achievement						Mathematics						Reading					
	*N*	#*ES*	*r*	*y*_i_	*v*_i_	*w*_i_	*N*	#*ES*	*r*	*y*_i_	*v*_i_	*w*_i_	*N*	#*ES*	*r*	*y*_i_	*v*_i_	*w*_i_
Al-Hendavi, 2010 [115]	72	1	0.31	0.32	0.01	1.98												
Blair and Razza, 2007 [33]	170	6	0.22	0.22	0.01	2.53	170	2	0.27	0.27	0.01	3.78	170	4	0.19	0.19	0.01	3.55
Bruni et al., 2006 [116]	264	1	0.65	0.78	0.00	2.72												
Bryce et al., 2018 [117]	210	2	0.19	0.19	0.01	2.63												
Checa and Abundis-Gutierrez, 2017 [92]	189	1	0.43	0.46	0.01	2.58												
Checa et al., 2008 [118]	61	2	0.50	0.56	0.02	1.85	61	2	0.45	0.49	0.01	2.40						
Chen et al., 2015 [54]	234	4	0.22	0.23	0.00	2.68	242	2	0.23	0.23	0.00	4.16	242	2	0.22	0.22	0.00	3.89
Chong et al., 2019 [119]	2621	2	0.07	0.07	0.00	3.10	2621	1	0.07	0.07	0.00	5.30	2621	1	0.07	0.07	0.00	4.88
Dindo et al., 2017 [121]	215	4	0.21	0.21	0.01	2.64	215	2	0.15	0.15	0.01	4.04	215	2	0.27	0.28	0.01	3.78
Fox et al., 2001–2010 [122]	145	2	0.22	0.22	0.01	2.45	146	1	0.20	0.21	0.01	3.59	144	1	0.23	0.30	0.01	3.36
Gaias et al., 2016 [123]	174	2	0.38	0.39	0.01	2.54	174	1	0.38	0.40	0.01	3.81	174	1	0.37	0.39	0.01	4.37
Galian et al., 2018 [124]													472	1	0.32	0.33	0.00	3.57
Gullesserian, 2009 [125]	29	5	−0.10	−0.10	0.04	1.23	29	1	−0.12	−0.12	0.04	1.42	29	2	−0.08	−0.08	0.04	1.37
Han et al., 2017 [127]													220	1	0.06	0.06	0.01	3.80
Hegvik, 1985 [93]	50	4	0.51	0.56	0.02	1.69							50	2	0.56	0.63	0.02	2.03
Hernandez, 2002 [128]	114	14	0.12	0.13	0.01	2.31	114	6	0.07	0.07	0.01	3.27	114	8	0.16	0.17	0.01	3.09
Hirvonen et al., 2013 [129]	152	2	0.34	0.35	0.01	2.47	152	1	0.29	0.30	0.01	3.64	152	1	0.38	0.40	0.01	3.42
Hirvonen et al., 2019 [130]	659	1	0.52	0.58	0.00	2.96												
Huang and Yeh, 2019 [47]													72	3	0.25	0.25	0.01	2.51
Iyer et al., 2010 [132]	394	3	0.33	0.34	0.00	2.85												
Johns et al., 2019 [58]	292	3	0.27	0.27	0.00	2.76	290	1	0.30	0.31	0.00	4.33	293	2	0.25	0.25	0.00	4.05
Kornienko et al., 2018 [134]	614	5	0.28	0.29	0.00	2.95												
Liew et al., 2008 [34]	677	10	0.12	0.12	0.00	2.97	677	5	0.16	0.16	0.00	4.91	677	5	0.09	0.09	0.00	4.54
Liu et al., 2018 [37]	184	2	0.09	0.09	0.01	2.57	184	1	0.06	0.06	0.01	3.87	184	1	0.12	0.12	0.01	3.63
Marcynyszyn, 2006 [135]	80	3	0.15	0.15	0.01	2.06	80	1	0.20	0.20	0.01	2.78	80	2	0.12	0.12	0.01	2.64
Martin and Holbrook, 1985 [28]	104	8	0.52	0.58	0.10	2.25	104	4	0.50	0.55	0.01	3.15	104	4	0.54	0.61	0.01	2.98
Martin et al., 1988, Study 1 [11]	82	10	0.48	0.53	0.01	2.08	90	6	0.42	0.45	0.01	2.95	85	8	0.43	0.47	0.01	2.72
Martin et al., 1988, Study 2 [11]	22	14	0.31	0.32	0.05	1.01	22	4	0.38	0.40	0.05	1.12	22	10	0.28	0.29	0.05	1.08
Martin et al., 1988, Study 3 [11]	63	8	0.36	0.38	0.02	1.88	63	4	0.41	0.43	0.02	2.45	63	4	0.32	0.33	0.02	2.33
Miller, 1999 [95]	141	24	0.03	0.03	0.01	2.43	140	12	0.03	0.03	0.01	3.54	142	12	0.04	0.04	0.01	3.35
Moreira et al., 2012 [136]	198	2	0.27	0.27	0.01	2.61												
Morris et al., 2013 [137]	74	2	0.43	0.45	0.01	2.00	74	1	0.42	0.45	0.01	2.67	74	1	0.43	0.46	0.01	2.54
Mullola et al., 2014 [60]							636	2	0.38	0.40	0.00	4.88	427	2	0.38	0.40	0.00	4.31
Oliver et al., 2007 [138]	103	2	0.46	0.49	0.01	2.24	52	2	0.26	0.27	0.02	2.18	52	2	0.23	0.24	0.02	2.08
Palisin, 1986 [139]	50	4	0.21	0.21	0.02	1.69												
Raymo et al., 2019 [140]	291	1	0.42	0.45	0.00	2.76												
Razza et al., 2012 [45]	2595	4	0.21	0.22	0.00	3.10	2595	2	0.22	0.22	0.00	5.29	2595	2	0.21	0.21	0.00	4.88
Sanchez-Perez et al., 2018 [35]	142	12	0.25	0.25	0.01	2.44	142	6	0.24	0.25	0.01	3.56	142	6	0.27	0.28	0.01	3.35
Studer-Luethi et al., 2016 [141]	99	2	0.25	0.26	0.01	2.21	99	1	0.29	0.30	0.01	3.08	99	1	0.21	0.21	0.01	2.92
Swanson et al., 2014 [89]							230	6	0.28	0.29	0.00	4.11						
Valiente et al., 2013 [49]	191	8	0.51	0.56	0.01	2.59												
Valiente et al., 2014 [90]	278	12	0.28	0.29	0.00	2.74												
Wang et al., 2017, Study 1 [50]	429	9	0.07	0.07	0.00	2.87	429	3	0.04	0.04	0.00	4.64	429	6	0.08	0.08	0.00	4.31
Wang et al., 2017, Study 2 [50]	1016	6	0.25	0.26	0.00	3.02	1021	3	0.23	0.23	0.00	5.08	1022	3	0.27	0.28	0.00	4.69
Zhou et al. 2010 [142]	404	6	0.29	0.30	0.00	2.86												
Zorza et al. 2019 [143]	244	1	0.43	0.46	0.00	2.69												

EC = effortful control; NA = negative affectivity; SU = surgency; *N* = sample size; #*ES* = number of effect sizes, from which one effect size was obtained; ES*_r_* = row effect size; *y*_i_ = transformed effect size; *v*_i_ = sampling variance; *w*_i_ = weight (the values are given in percent).

**Table A2:** List of studies with individual effect sizes: Negative affectivity and academic achievement.

Author(s), Year	Overall Achievement						Mathematics						Reading					
	*N*	#*ES*	*r*	*y*_i_	*v*_i_	*w*_i_	*N*	#*ES*	*r*	*y*_i_	*v*_i_	*w*_i_	*N*	#*ES*	*r*	*y*_i_	*v*_i_	*w*_i_
Al-Hendavi, 2010 [115]	72	2	−0.11	−0.11	0.01	1.84												
Bruni et al., 2006 [116]	264	1	−0.21	−0.21	0.00	3.98												
Bryce et al., 2018 [117]	210	2	0.03	0.03	0.01	3.59												
Checa and Abundis-Gutierrez, 2017 [92]	189	1	−0.22	−0.22	0.01	3.41												
Checa et al., 2008 [118]	61	2	−0.23	−0.24	0.02	1.62	61	2	−0.21	−0.22	0.01	1.92						
Chong et al., 2019 [119]	2621	2	−0.10	−0.10	0.00	6.36	2621	1	−0.09	−0.09	0.00	16.21	2621	1	−0.11	−0.11	0.00	9.28
Colom et al., 2007 [120]	135	1	−0.24	−0.25	0.01	2.83	135	1	−0.26	−0.27	0.01	3.87	135	2	−0.30	−0.31	0.01	4.20
Fox et al., 2001–2010 [122]	145	1	−0.06	−0.06	0.01	2.95	146	1	−0.07	−0.07	0.01	4.13	144	1	−0.05	−0.05	0.01	4.36
Gaias et al., 2016 [123]	174	2	−0.26	−0.27	0.01	3.26	174	1	−0.23	−0.23	0.01	4.74	174	1	−0.29	−0.30	0.01	4.84
Gullesserian, 2009 [125]	29	5	0.09	0.09	0.04	0.84	29	1	0.24	0.25	0.04	0.91	29	2	−0.02	−0.02	0.04	1.26
Gumora and Arsenio, 2002 [126]	103	4	−0.17	−0.18	0.01	2.38												
Han et al., 2017 [127]													220	1	−0.01	−0.01	0.01	5.43
Hegvik, 1985 [93]	50	12	−0.21	−0.22	0.02	1.38	50	1	−0.42	−0.45	0.02	1.58	50	3	−0.19	−0.19	0.02	2.06
Hernandez, 2002 [128]	114	14	−0.15	−0.15	0.01	2.55	114	6	−0.14	−0.14	0.01	3.36	114	8	−0.15	−0.15	0.01	3.79
Hirvonen et al., 2013 [129]	152	4	−0.13	−0.13	0.01	3.03	152	2	−0.11	−0.11	0.01	4.26	152	2	−0.16	−0.16	0.01	4.50
Hirvonen et al., 2019 [130]	659	1	−0.27	−0.28	0.00	5.31												
Hsieh, 1998 [131]	230	2	−0.06	−0.06	0.00	3.75												
Huang and Yeh, 2019 [47]													72	3	−0.03	−0.03	0.01	2.75
Jeronimus et al., 2015 [133]	1534	2	−0.05	−0.05	0.00	6.08												
Johns et al., 2019 [58]	292	1	−0.16	−0.17	0.00	4.15	290	1	−0.16	−0.16	0.00	6.83	293	2	−0.17	−0.17	0.00	6.13
Kwon et al., 2018 [62]													199	3	−0.21	−0.22	0.01	5.18
Liu et al., 2018 [37]	184	2	−0.07	−0.07	0.01	3.36	184	1	−0.09	−0.09	0.01	4.95	184	1	−0.05	−0.05	0.01	4.98
Martin and Holbrook, 1985 [28]	104	8	−0.35	−0.37	0.01	2.40	104	4	−0.33	−0.34	0.01	3.11	104	4	−0.38	−0.39	0.01	3.57
Martin et al., 1988, Study 1 [11]	82	10	−0.30	−0.31	0.01	2.03	90	6	−0.20	−0.20	0.01	2.74	85	8	−0.27	−0.28	0.01	3.11
Martin et al., 1988, Study 2 [11]	22	14	−0.10	−0.10	0.05	0.63	22	4	−0.09	−0.09	0.05	0.67	22	10	−0.10	−0.10	0.05	0.95
Martin et al., 1988, Study 3 [11]	63	8	0.00	0.00	0.02	1.66	63	4	0.00	0.00	0.02	1.98	63	4	0.00	0.00	0.02	2.49
Miller, 1999 [95]	141	24	−0.03	−0.03	0.01	2.90	140	12	−0.05	−0.05	0.01	3.99	142	12	−0.02	−0.02	0.01	4.33
Mullola et al., 2014 [60]							636	2	−0.16	−0.16	0.00	10.59	427	2	−0.19	−0.19	0.00	6.97
Oades-Sese et al., 2011 [87]	149	14	−0.23	−0.23	0.01	2.30	90	2	−0.29	−0.29	0.01	2.74	149	12	−0.22	−0.23	0.01	4.45
Ooi et al., 2017 [88]	150	1	−0.05	−0.05	0.01	3.01												
Palisin, 1986 [139]	50	4	−0.01	−0.01	0.02	1.38												
Scrimin et al., 2019 [48]	91	1	−0.01	−0.01	0.01	2.19												
Talwar et al., 1989 [30]	150	3	−0.31	−0.32	0.01	3.01												
Wang et al., 2017, Study 1 [50]	429	9	−0.05	−0.05	0.00	4.74	429	3	−0.08	−0.08	0.00	8.67	429	6	−0.04	−0.04	0.00	6.98
Wang et al., 2017, Study 2 [50]	1009	6	−0.05	−0.05	0.00	5.75	1008	3	−0.06	−0.06	0.00	12.75	1009	3	−0.04	−0.04	0.00	8.42
Zhou et al. 2010 [142]	404	6	−0.11	−0.11	0.00	4.66												

EC = effortful control; NA = negative affectivity; SU = surgency; *N* = sample size; #*ES* = number of effect sizes, from which one effect size was obtained; ES*_r_* = row effect size; *y*_i_ = transformed effect size; *v*_i_ = sampling variance; *w*_i_ = weight (the values are given in percent).

**Table A3:** List of studies with individual effect sizes: Surgency and academic achievement.

Author(s), Year	Overall Achievement						Mathematics						Reading					
	*N*	#*ES*	*r*	*y*_i_	*v*_i_	*w*_i_	*N*	#*ES*	*r*	*y*_i_	*v*_i_	*w*_i_	*N*	#*ES*	*r*	*y*_i_	*v*_i_	*w*_i_
Al-Hendavi, 2010 [115]	72	1	−0.27	−0.28	0.01	2.74												
Bruni et al., 2006 [116]	264	1	0.40	0.42	0.00	3.83												
Bryce et al., 2018 [117]	210	2	−0.05	−0.05	0.01	3.70												
Checa et al., 2008 [118]	61	2	0.03	0.03	0.02	2.56	61	2	0.13	0.13	0.02	3.06						
Chong et al., 2019 [119]	2621	2	0.03	0.03	0.00	4.40	2621	1	0.02	0.02	0.00	10.12	2621	1	0.04	0.04	0.00	11.08
Colom et al., 2007 [120]	135	1	−0.30	−0.03	0.01	3.36	135	2	−0.12	−0.12	0.01	5.11	135	4	−0.25	−0.25	0.01	4.56
Fox et al., 2001–2010 [122]	145	1	−0.11	−0.11	0.01	3.42	146	1	−0.08	−0.08	0.01	5.32	144	1	−0.14	−0.14	0.01	4.74
Gaias et al., 2016 [123]	174	2	0.03	0.03	0.01	3.57	174	1	−0.29	−0.30	0.01	5.79	174	1	0.05	0.05	0.01	5.31
Gullesserian, 2009 [125]	29	5	0.03	0.03	0.04	1.67	29	1	−0.04	−0.04	0.04	1.63	29	2	0.19	0.19	0.04	1.29
Gumora and Arsenio, 2002 [126]	103	2	0.29	0.30	0.01	3.12												
Han et al., 2017 [127]													220	2	−0.02	−0.02	0.01	6.02
Hegvik, 1985 [93]	50	4	0.41	0.44	0.02	2.32							50	1	0.41	0.44	0.02	2.15
Hernandez, 2002 [128]	114	14	−0.02	−0.02	0.01	3.21	114	6	0.00	0.00	0.01	4.65	114	8	−0.06	−0.06	0.01	4.08
Hirvonen et al., 2013 [129]	152	2	0.02	0.02	0.01	3.46	152	1	0.03	0.03	0.01	5.43	152	1	0.01	0.01	0.01	4.90
Hsieh, 1998 [131]	230	1	0.04	0.04	0.00	3.75												
Hughes and Coplan, 2010 [94]	125	2	−0.18	−0.18	0.01	3.30	125	1	−0.22	−0.23	0.01	4.90	125	1	−0.14	−0.14	0.01	4.34
Liu et al., 2018 [37]	184	2	−0.08	−0.08	0.01	3.61	184	1	−0.05	−0.05	0.01	5.95	184	1	−0.11	−0.11	0.01	5.48
Martin and Holbrook, 1985 [28]	104	8	0.00	0.00	0.01	3.13	104	4	0.03	0.03	0.01	4.40	104	4	−0.03	−0.03	0.01	3.83
Martin et al., 1988, Study 1 [11]	82	10	−0.18	−0.19	0.01	2.88	90	6	−0.26	−0.26	0.01	4.02	85	8	−0.13	−0.13	0.01	3.31
Martin et al., 1988, Study 2 [11]	22	14	0.17	0.17	0.05	1.36	22	4	0.10	0.10	0.05	1.24	22	10	0.19	0.19	0.05	0.97
Martin et al., 1988, Study 3 [11]	63	8	−0.07	−0.07	0.02	2.59	63	4	0.08	0.08	0.02	3.14	63	4	−0.21	−0.22	0.02	2.61
Miller, 1999 [95]	141	24	0.04	0.04	0.01	3.40	140	12	0.06	0.06	0.01	5.20	142	12	0.02	0.02	0.01	4.70
Moreira et al., 2012 [136]	198	2	−0.11	−0.11	0.01	3.66												
Mullola et al., 2014 [60]							636	2	−0.06	−0.06	0.00	8.70	427	2	−0.01	−0.01	0.00	7.95
Oades-Sese et al., 2011 [87]	149	7	−0.22	−0.23	0.01	3.45	90	1	−0.25	−0.26	0.01	4.02	149	6	−0.22	−0.22	0.01	4.84
Ooi et al., 2017 [88]	150	1	0.15	0.15	0.01	3.45												
Palisin, 1986 [139]	50	7	−0.06	−0.06	0.02	2.32												
Scrimin et al., 2019 [48]	91	3	0.21	0.22	0.01	2.99												
Talwar et al., 1989 [30]	150	3	−0.12	−0.12	0.01	3.45												
Valiente et al., 2013 [49]	191	8	−0.19	−0.19	0.01	3.63												
Wang et al., 2017, Study 1 [50]	429	9	−0.02	−0.02	0.00	4.06	429	3	0.06	0.06	0.00	7.98	429	6	−0.05	−0.05	0.00	7.96
Wang et al., 2017, Study 2 [50]	1015	6	−0.01	−0.01	0.00	4.29	1018	3	−0.02	−0.02	0.00	9.35	1019	3	0.01	0.01	0.00	9.89
Zhang et al., 2017 [91]	128	3	0.23	0.23	0.01	3.32												

EC = effortful control; NA = negative affectivity; SU = surgency; *N* = sample size; #*ES* = number of effect sizes, from which one effect size was obtained; ES*_r_* = row effect size; *y*_i_ = transformed effect size; *v*_i_ = sampling variance; *w*_i_ = weight (the values are given in percent.
